# Treatment of Patent Ductus Arteriosus in Premature Infants: Intravenous Paracetamol or Oral Ibuprofen?

**DOI:** 10.34172/aim.2023.50

**Published:** 2023-06-01

**Authors:** Naeeme Taslimi Taleghani, Banafshe Hamrahi, Minoo Falahi, Eisa Nazar, Farzane Palizban, Ali Naseh, Maryam Khoshnood Shariati

**Affiliations:** ^1^Neonatal Health Research Center, Research Institute for Children’s Health, Shahid Beheshti University of Medical Sciences, Tehran, Iran; ^2^Clinical Research Development Center, Mahdiyeh Educational Hospital, Shahid Beheshti University of Medical Sciences, Tehran, Iran; ^3^Psychiatry and Behavioral Sciences Research Center, Addiction Institute, Mazandaran University of Medical Sciences, Sari, Iran

**Keywords:** Acetaminophen, Adverse effects, Ibuprofen, Paracetamol, Patent ductus arteriosus

## Abstract

**Background:**

The similarity in the mechanism of action between paracetamol and ibuprofen can cause similar side effects. However, in preterm neonates with feeding intolerance, intravenous (IV) paracetamol has replaced oral ibuprofen. Therefore, a comparison of the effectiveness and side effects is essential.

**Methods:**

In this retrospective cohort study, the data of 118 preterm infants with a definite diagnosis of patent ductus arteriosus (PDA), including 59 patients who received oral ibuprofen and 59 patients who received IV paracetamol were analyzed. Laboratory evaluations of serum total and direct bilirubin, hemoglobin, and creatinine levels before and seven days after treatment were made. Using analysis of covariance (ANCOVA) and multiple multinomial logistic regression models, the effect of two treatment groups on the post-treatment variables as well as their efficacy comparison were evaluated.

**Results:**

In both pre- and post-treatment periods, there was no significant association between echocardiography variables with treatment groups. The results from the ANCOVA model showed that the paracetamol and ibuprofen were followed by a significant decrease in the mean total bilirubin and Hct variables after treatment by 1.38 and 1.65 units, respectively. In addition, results from the Mann-Whitney U test revealed that the median Hb and K differences after and before treatment had a significant difference between the two treatment groups. Furthermore, based on the multiple multinomial logistic model results, the odds of complete arterial duct closure with IV paracetamol was 1.27 times higher than with oral ibuprofen, while in the oral ibuprofen group, the odds of closing was 1.44 times higher than the IV paracetamol group, but there was no statistically significant difference between the two groups.

**Conclusion:**

Intravenous paracetamol has equal efficacy compared to oral ibuprofen in the treatment of PDA. Also, it seems to be associated with a lower risk of hyperbilirubinemia following the treatment.

## Introduction

 Ductus arteriosus is a vascular structure connecting the proximal part of the descending aortic artery to the main pulmonary artery near the site of detachment of the left pulmonary artery during embryonic development. This important embryonic structure is normally closed in the first few days after birth; if left open, it can lead to a patent ductus arteriosus (PDA).^1^ PDA is one of the most common neonatal anomalies, occurring in very premature infants at about 30%-67%.^2^ This condition leads to a blood shunt from descending aorta to the pulmonary artery (i.e. left-to-right shunt). This shunt can lead to enlarged heart cavities and increased pulmonary artery pressure, so it is potentially fatal.^3,4^ Therefore, it seems PDA treatment is helpful to prevent pulmonary hypertension and improve the cardio-respiratory status survival.^5^ Furthermore, PDA can increase the duration of use of mechanical ventilation support, the risk of morbidities (such as necrotizing enterocolitis and chronic lung diseases), and mortality.^6,7^ The spontaneous closure of PDA is rare after a few weeks. Surgical treatment is recommended if medication therapy fails, although it may be potentially fatal for the extremely preterm neonate.^1,8^ The ductus arteriosus spontaneously closes in only about 30% of infants weighing less than 1000 g.^4,9^ Many experts believe that closure of PDA should be considered only in cases of hemodynamically significant PDA. Nevertheless, description of the hemodynamically significant inhibitory artery is ambiguous and there is no universal agreement on it. There are several clinical, echocardiographic, and other related variables that should be considered when defining hemodynamically significant patent ductus arteriosus (hsPDA). Echocardiographic indicators can be classified into the following for evaluating PDA as hsPDA: PDA shunt size, volume overload, pulmonary degree overflow, and the rate of systemic hypoperfusion.^10,11^ The conventional treatment is through non-steroidal anti-inflammatory drugs (NSAIDs), especially indomethacin and ibuprofen.^3,4^ Ibuprofen is preferable to indomethacin in the treatment of PDA due to its lower side effects.^12,13^ Another drug is paracetamol (acetaminophen) which has been used to treat PDA since 2011.^14^ Paracetamol is better known as an antipyretic and analgesic. Acetaminophen affects the peroxidase site of the prostaglandin H synthase (PGHS) enzyme; by directly inhibiting the function of this enzyme, it reduces the level of prostaglandins.^15^ Given its less vasoconstriction, paracetamol has fewer known side effects than ibuprofen.^12,16-18^ Acetaminophen has the least reducing action in inflammatory sites because the concentration of the oxidant agent is high there. So when it is used as an anti-inflammatory and analgesia drug, it has less activity in the periphery than in the brain due to the concentration of oxidant agents. This can explain why it has fewer peripheral adverse effects (gastric upset and platelet inhibition) than NSAIDs.^19^ However, because of paracetamol’s mechanism of action, the presence of comparable side effects to NSAID is possible.^20^ Newborns and infants who have received an overdose of paracetamol are at low risk of serious hepatic damage, while those who have recently ingested more than one supra therapeutic dose of paracetamol should be managed with caution for hyperchloremic metabolic acidosis, hypokalemia, and bone marrow suppression.^21^ Gastrointestinal bleeding or perforation, acute renal failure, and multi-organ dysfunction have been reported in ibuprofen overdose.^22,23^ Although many studies have compared the efficacy of paracetamol and ibuprofen, possible side effects such as hyperbilirubinemia, anemia, renal failure, or GI upset among preterm infants have been less compared.^3,9,15,18,24,25^ Also, some studies have reported certain neurological impairments at 7 and 11 years of age in those who were in contact with acetaminophen in the early neonatal or fetal life.^26^ Given the fact that there are still many questions about the safety of administration of intravenous (IV) acetaminophen in short- and long-term, the importance of such studies is also prominent.^18,22^ In our NICU, decisions on PDA treatment are based on a set of clinical signs and echocardiographic findings. Some considered clinical manifestations include increased need for respiratory support, inability to wean from mechanical ventilation, manifestations of pulmonary edema or congestion, or hemorrhage due to PDA. In our country, all centers do not have access to neonatal type IV ibuprofen due to its high cost; therefore, in the absence of feeding tolerance, IV paracetamol (acetaminophen) has been widely welcomed. So the reason for using any of these drugs in our NICU is their availability and also, the status of neonatal feeding tolerance. The purpose of the current study is to assess the efficacy of treatment and compare the prevalence of side effects such as hyperbilirubinemia and transient renal function impairment in preterm infants with IV paracetamol and oral ibuprofen. As far as we were able to verify, until the time of this research, there was no study to examine the difference in effectiveness and possible side effects for IV paracetamol and oral ibuprofen.

## Materials and Methods

###  Study Population

 The study was performed as a retrospective cohort study to evaluate the data of 118 preterm infants with PDA who were admitted to the NICU in a tertiary center. We included 59 patients who were treated with oral ibuprofen and 59 patients who were treated with IV paracetamol as first line treatment after consent was obtained from their parents. The reason for using any of these drugs was their availability and also, the status of neonatal feeding tolerance.

###  Inclusion and Exclusion Criteria

 Inclusion criteria were as follows: preterm infants in the first two weeks of life in whom PDA was diagnosed by clinical examinations and echocardiographic confirmation who took IV paracetamol at a dose of 15 mg/kg every 6 hours for 3 days or oral ibuprofen at a first dose of 10 mg/kg and then 5 mg/kg at 24-hour intervals for 2 days. The considered echocardiographic confirmatory findings included: left atrial dilatation (i.e. left atrial to aortic root ratio greater than 1.6), diastolic turbulence (i.e. reversal flow) in the pulmonary artery, internal duct diameter greater than 1.5 mm, reversal flow in the descending aortic artery and mesenteric artery and at the end of diastole in echocardiography. In the current study, infants with congenital anomalies or any significant genetic conditions, life-threatening sepsis, necrotizing enterocolitis, intraventricular hemorrhage, and platelet counts less than 100 000/ µL were excluded.

###  Procedure

 In this study, two groups were compared in terms of the frequency of PDA closure. The diagnosis of PDA closure was confirmed by re-echocardiography and taking into account the above-mentioned criteria. At the time of each echocardiography of the neonates, the patency or closure of ductus arteriosus was determined based on eight parameters (PP, HR, A/MV, FP, SM, HM, AC, PD). Laboratory evaluations of total and direct bilirubin levels and hemoglobin and creatinine levels before and after treatment with the mentioned medicines were made using colorimetric kits (Sigma-Aldrich, USA). Based on our NICU’s practical protocol, the neonates’ bilirubin level, creatinine levels, and hemoglobin were recorded before treatment and also after seven days. Seven days after starting treatment, neonates underwent echocardiography and were classified and evaluated for the patency or closure of ductus arteriosus.

###  Statistical Analysis

 Mean ± SD and frequency (%) were computed to report the quantitative and qualitative variables, respectively. In order to measure the association between qualitative variables, Chi-square test was applied and if more than 20% of the expected counts (Eij) were less than 5, we used Fisher’s exact test instead of chi-square. In addition, the mean values of normal quantitative variables between the two treatment groups were compared using an independent T test and if the normality assumption was violated, the Mann-Whitney U test was used to compare the median of quantitative variables between the two treatment groups. Furthermore, an analysis of covariance (ANCOVA) model was applied to compare the effect of the two treatments (ibuprofen & paracetamol) on the response variables including total bilirubin, direct bilirubin, white blood cell (WBC), neutrophil, lymphocyte, Hb, Hct, retic, blood urea nitrogen (BUN), creatinine (Cr), Na, and K by controlling the effects of pre-treatment measurements. It should be noted that if one of the ANCOVA model assumptions including the normality, homogeneity of variance, and no interaction assumptions, was violated, the results of Mann-Whitney U test were reported instead of the ANCOVA model for differences of responses between before and after treatment. Also, the efficacy of the two treatments including three therapeutic responses (no treatment response, closing, and closed) with adjusting the effect of confounders, was compared by a multiple multinomial logistic regression model. Thus, variables with *P* < 0.30 in the univariate model were entered into the multiple model. All analyses were performed in the SPSS version 20.0 at the significance level of 0.05.

## Results

**Figure 1:**
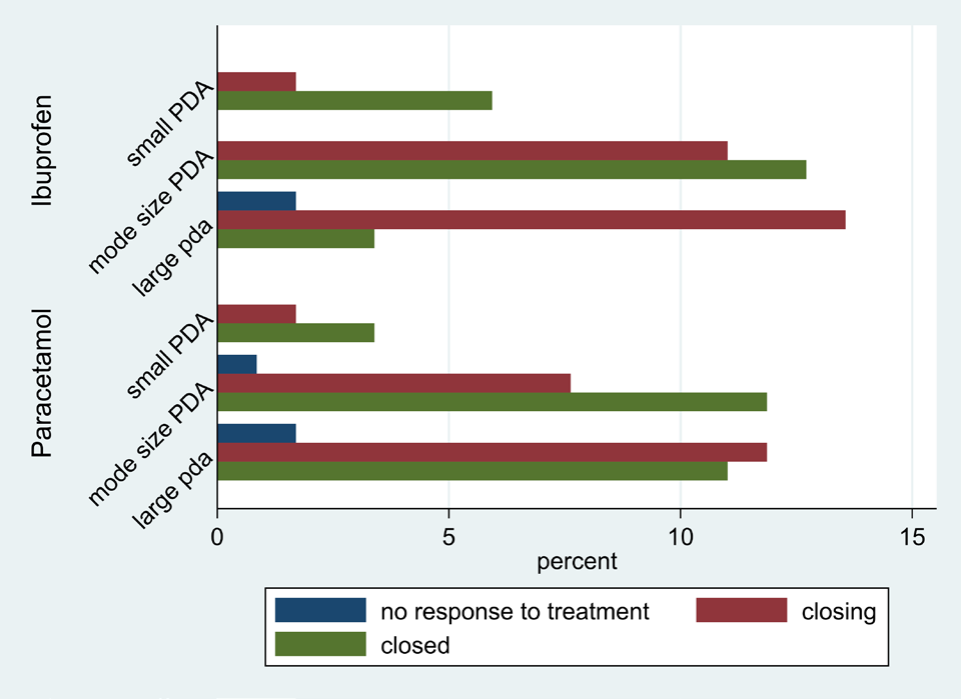
Percentage Frequency Distribution of Treatment Outcomes by Group and Echocardiography Result Before Treatment

 A total of 118 preterm infants with PDA were included, of whom 59 neonates were treated with IV paracetamol and 59 with oral ibuprofen. The mean birth weight in the ibuprofen group was higher compared to the paracetamol group (1355.76 ± 414.21 vs. 1330.33 ± 379.14, respectively); however, the difference in mean birth weight between the two groups was not statistically significant. Also, there was no statistically significant difference in the mean gestational age between the two groups. In addition, there was no significant difference in the frequency distribution of gender between the two groups. As shown in Table 1, the frequency distribution of other variables also did not have a statistically significant difference between the two groups. Therefore, the two treatment groups were homogeneous in terms of weight, gestational age, gender and other demographic variables. In both pre- and post-treatment period, there was no significant association between echocardiography variables and treatment groups. In other words, there was no significant difference in the frequency distribution of echocardiography variables in the two groups of ibuprofen and paracetamol (Table 2). Figure 1 shows that the neonates with a small PDA had a successful therapeutic response (closed or closing outcomes).

**Table 1:** Comparison of Demographic and Clinical Characteristics of Patients Between the Ibuprofen and Paracetamol Groups

**Characteristics**	**Total**	**Group**		***P***** Value**
		**Ibuprofen**	**Paracetamol **	
Gender				0.99
Female	82 (100)	41 (50.00)	41 (50.00)	
Male	36 (100)	18 (50.00)	18 (50.00)	
Pregnancy outcome				0.65
Singleton	92 (100)	47 (51.10)	45 (48.90)	
Multiple birth	26 (100)	12 (46.20)	14 (53.80)	
Reproductive assist methods				0.48
No	95 (100)	49 (51.60)	46 (48.40)	
Yes	23 (100)	10 (43.50)	13 (56.50)	
Parity				0.58
Primipara	57 (100)	30 (52.60)	27 (47.40)	
Multipara	61 (100)	29 (47.50)	32 (52.50)	
Delivery				0.54
NVD	27 (100)	12 (44.40)	15 (55.60)	
C/S	90 (100)	46 (51.10)	44 (48.90)	
Weight (g)	1453.64 ± 1274.19	1355.76 ± 414.21	1330.33 ± 379.14	0.72
Gestational age	30.56 ± 2.34	30.81 ± 2.32	30.32 ± 2.35	0.26

NVD, normal vaginal delivery; C/S, Cesarean section.

**Table 2:** Comparison of Clinical Characteristics Between the Two Groups (Ibuprofen & Paracetamol) by Treatment Period

**Characteristics** **Echocardiography**^a^	**Treatment period**							
	**Before**				**After**			
	**Total**	**Group**		***P***	**Total**	**Group**		***P***
		**Ibuprofen**	**Paracetamol**			**Ibuprofen**	**Paracetamol**	
Small size PDA	15 (100)	9 (60.00)	6 (40.00)	0.39	5 (100)	2 (40.00)	3 (60.00)	0.58
Mode size PDA	52 (100)	28 (53.80)	24 (46.20)		56 (100)	31 (55.40)	25 (44.60)	
Large size PDA	51 (100)	22 (43.10)	29 (56.90)		57 (100)	26 (45.60)	31 (54.40)	

^a^Levels of echocardiography variable after treatment include no response to treatment (5 cases), closing (56 cases), and closed (57 cases).

 The results of independent t-test and Mann-Whitney U test are presented in Table 3. According to independent t-test in the pre-treatment period, the mean values of none of the total bilirubin, WBC, neutrophil, lymphocyte, Hct, and Cr variables did not show a statistically significant difference between the two treatment groups. Also, the results of the Mann-Whitney U test in the pre-treatment period revealed that the median values of BUN (median of ibuprofen and paracetamol groups: 15.00 vs. 18.00) and K (median of ibuprofen and paracetamol groups: 4.60 vs. 4.20) showed a significant difference between the two groups. Furthermore, in the post-treatment stage, a significant difference was observed in the mean values of total bilirubin (mean of ibuprofen and paracetamol groups: 9.66 vs. 8.28) and hemoglobin (mean of ibuprofen and paracetamol groups: 13.25 vs. 12.69) between the two groups based on the results of independent t-test. At this stage, other variables were not statistically significantly different between the two groups.

**Table 3:** Comparison of the mean of quantitative clinical variables between the two groups of ibuprofen and paracetamol by treatment period

**Characteristics**	**Treatment period**							
	**Before**				**After**			
	**Total**	**Group**		***P***	**Total**	**Group**		***P***
		**Ibuprofen**	**Paracetamol**			**Ibuprofen**	**Paracetamol**	
Total bilirubin	8.41 (2.06)	8.41 (1.61)	8.40 (2.44)	0.98	8.96 (2.15)	9.66 (2.16)	8.28 (1.93)	< 0.001
Direct bilirubin	0.40 (0.40-0.40)	0.40 (0.40-0.40)	0.40 (0.40-0.40)	0.72	0.40 (0.30-0.40)	0.40 (0.30-0.40)	0.40 (0.30-0.40)	0.19
WBC	13548.56 (4341.94)	13272.54 (3862.32)	13824.58 (4791.38)	0.49	11650.00 (9200-14000)	12000.00 (9250-14000)	11500.00 (9000-14000)	0.65
Neutrophil	54.69 (14.76)	56.89 (14.70)	52.50 (14.62)	0.10	52.54 (10.77)	53.84 (10.13)	51.24 (11.31)	0.19
Lymphocyte	41.36 (12.96)	39.73 (13.52)	42.98 (12.26)	0.17	43.16 (9.40)	42.74 (9.71)	43.59 (9.13)	0.62
Hb	14.50 (13.77-15.82)	14.50 (13.80-15.50)	14.50 (13.60-16.00)	0.62	12.97 (1.52)	13.25(1.24)	12.69 (1.73)	0.04
Hct	43.61 (5.70)	42.82 (4.85)	44.40 (6.39)	0.13	38.97 (4.33)	39.38 (3.69)	38.56 (4.89)	0.31
Retic (%)	3.00 (2.00-4.00)	3.00 (2.00-4.00)	3.00 (2.00-4.50)	0.39	2.00 (1.20-3.00)	2.00 (1.50-3.00)	2.00 (1.20-3.00)	0.72
BUN	17.00 (14.00-20.00)	15.00 (14.00-18.00)	18.00 (14.00-24.00)	0.03	14.30 (12.00-18.00)	14.00 (12.00-17.00)	15.00 (12.00-18.00)	0.38
Cr	0.83 (0.21)	0.80 (0.19)	0.85 (0.23)	0.21	0.70 (0.60-0.80)	0.70 (0.60-0.80)	0.70 (0.60-0.80)	0.12
Na	140.00 (139.00-143.25)	141.00 (140.00-143.00)	140.00 (139.00-145.00)	0.56	140.18 (3.62)	140.69 (3.70)	139.67 (3.50)	0.12
K	4.30 (3.97-4.82)	4.60 (4.00-4.90)	4.20 (3.90-4.50)	0.002	4.10 (3.97-4.80)	4.20 (3.90-5.00)	4.10 (4.00-4.70)	0.29

Hb, Hemoglobin; Hct, Hematocrit; WBC, white blood count cells; BUN, blood urea nitrogen; Cr, Creatinine; Na, Sodium, Potassium. Values are reported as mean (SD) for independent t-test and median (Q1-Q3) for Mann-Whitney U test.

Table 4 shows the results of the ANCOVA model. It is worth noting that the ANCOVA model assumptions were satisfied for all responses presented in Table 4. According to the results of this model, the treatment group showed a significant difference in the rate of total bilirubin with -1.38 (-2.11, -0.64) and Hct with -1.65 (-2.80, -0.50) after treatment. In other words, the mean of these variables after treatment was significantly different between the two treatment groups, such that the mean total bilirubin and Hct responses after treatment in the paracetamol group were respectively 1.38 (-2.11, -0.64) and 1.65 (-2.80, -0.50) units less than the ibuprofen group. However, the treatment group did not substantially differ in the rate of other responses after treatment.

**Table 4:** Comparison of the Effect of Two Treatment Groups on the Response Variables by Controlling the Effects of Pre-treatment Measurements Using the ANCOVA Model

**Response variables**^#^		**Coefficients **	**95 CI%**	***P***** value**
Total bilirubin	Ibuprofen	0.23	(0.05, 0.41)	0.01
	Paracetamol	-1.38	(-2.11, -0.64)	< 0.001
Direct bilirubin	Ibuprofen	-0.02	(-0.18, 0.13)	0.72
	Paracetamol	-0.01	(-0.03,0.006)	0.14
WBC	Ibuprofen	0.23	(-0.05, 0.52)	0.11
	Paracetamol	-1137.13	(-3631.40, 1357.13)	0.36
Neutrophil	Ibuprofen	0.33	(0.21, 0.45)	< 0.001
	Paracetamol	-1.13	(-4.67, 2.41)	0.52
Lymphocyte	Ibuprofen	0.22	(0.09, 0.35)	0.001
	Paracetamol	0.12	(-3.19, 3.44)	0.94
Hct	Ibuprofen	0.53	(0.43, 0.63)	< 0.001
	Paracetamol	-1.65	(-2.80, -0.50)	0.005
Retic	Ibuprofen	-0.09	(-0.64, 0.45)	0.74
	Paracetamol	-5.38	(-13.85, 3.09)	0.21
Cr	Ibuprofen	0.19	(-0.13, 0.52)	0.23
	Paracetamol	0.01	(-0.12, 0.15)	0.86
Na	Ibuprofen	0.002	(-0.03, 0.03)	0.89
	Paracetamol	-1.02	(-2.34, 0.30)	0.12

Hct, Hematocrit; WBC, white blood count cells; Cr, Creatinine; Na, Sodium. ^#^post-treatment measurement.

 The results from the Mann-Whitney U test showed that the median of Hb (median of ibuprofen and paracetamol groups: -1.50 vs. -1.80) and K (median of ibuprofen and paracetamol groups: -0.30 vs. 0.10) after and before treatment showed a significant difference between the two treatment groups (Table 5).

**Table 5:** Comparison of the Median of Quantitative Variables Between the Two Groups of Ibuprofen and Paracetamol using Mann-Whitney U test

**Response**^a^	**Total**	**Group**		***P***** Value**
		**Ibuprofen**	**Paracetamol**	
Hb	-1.50 (-2.12 - -1.20)	-1.50 (-1.70 - -1.00)	-1.80 (-3.00 - -1.40)	0.002
BUN	-3.00 (-6.00 – 1.00)	-2.00 (-4.00 – 1.00)	-4.00 (-7.00 – 1.00)	0.17
K	-0.15 (-0.50 – 0.40)	-0.30 (-0.80 - 0.50)	0.10 (-0.30 – 0.30)	0.02

Hb, Hemoglobin; K, Potassium; BUN, blood urea nitrogen. ^a^Response variables = post-treatment measurements - pre-treatment measurements. Values are reported as median (Q1-Q3).

 The results from the univariate multinomial logistic regression model revealed that none of the variables, including the treatment group, had a significant impact on therapeutic response and efficacy, such that the odds of closing and closed outcomes compared to no treatment response outcome in the paracetamol vs. ibuprofen group were 0.53 and 0.79 times, respectively; however, the difference between the two groups was not statistically significant (results not shown). In addition, the results of multiple multinomial logistic regression model also indicated that in the treatment group, the result of echocardiography before treatment, did not have a statistically significant influence on therapeutic response and efficacy, such that the odds of closing and closed outcomes compared to no treatment outcome in the paracetamol vs. ibuprofen group were 0.69 (0.10, 4.68) and 1.27 (0.18, 8.89) times, respectively. Also, the odds of closing and closed outcomes compared to no treatment outcome in patients with large PDA vs. small and mode PDA in the result of echocardiography before treatment were 0.31 (0.03, 3.02) and 0.10 (0.01, 1.04) times, respectively. This means that the odds of closing and closed outcomes decrease with increasing PDA grade (Table 6).

**Table 6:** Comparing the Efficacy of Two Drugs with Adjusting the Effect of Confounders using Multiple Multinomial Logistic Regression Model (Closing and Closed vs. No Treatment Response)

**Characteristics (Reference)**	**Closing vs. No Treatment Response**		**Closed vs. No Treatment Response**	
	**Odds Ratio (95% CI)**	***P***	**Odds Ratio (95% CI)**	***P***
Echocardiography (small & mode PDA) Large PDA	0.31 (0.03, 3.02)	0.31	0.10 (0.01, 1.04)	0.055
Group (Ibuprofen) Paracetamol	0.69 (0.10, 4.68)	0.71	1.27 (0.18, 8.89)	0.80

PDA, Patent ductus arteriosus.

## Discussion

 Ductus arteriosus is a vascular structure that connects the proximal descending aorta to the roof of the main pulmonary artery near the origin of the left branch pulmonary artery. ^1^ Although it is recommended to close the hemodynamically significant PDA to reduce complications, due to lack of sufficient information for PDA treatment, different methods and guidelines are used to manage the patients.^3,12^ In addition to disagreements over treatment indications and the patient’s age for starting treatment, there is still debate about the drug of choice.^3,9,27^ The present study shows that the efficacy of oral ibuprofen and IV paracetamol in PDA closure is not significantly different as first line treatment. As the results in Table 6 show, the chance of complete arterial duct closure (closed PDA) with IV paracetamol is 1.27 times higher than with oral ibuprofen (95% CI: (0.18, 8.89)). Paracetamol was first used to treat hemodynamically significant PDA by Hammerman et al in 2011.^14^ In their study, they included five preterm neonates with hemodynamically significant PDA for whom NSAID treatment had contraindications or NSAID treatment was not successful. Although the study by Hammerman et al introduced a new approach to the treatment of PDA, they could not provide an accurate assessment of drug efficacy due to the small sample size.^14^ Oncel et al used an IV paracetamol treatment similar to the present study and confirmed the results of the study by Hammerman et al.^28^ Unlike the present study, their study had only one treatment group and could not compare the efficacy of treatment by IV paracetamol to other drugs. In 2014, Oncel et al designed a randomized controlled trial to compare the effects of oral paracetamol and oral ibuprofen on the treatment of hemodynamically significant PDA. Their results showed that oral paracetamol and oral ibuprofen improved the closure in 72.5% and 77.5% of PDA neonates, respectively, but the efficacy of the two drugs did not differ significantly.^29^ In a recent systematic review and meta-analysis, Xiao et al reported no significant difference in efficacy between paracetamol and ibuprofen in PDA treatment. However, the mean number of days for PDA closure was smaller in the paracetamol group.^18^ Prolonged hyperbilirubinemia in preterm neonates is a common problem that is a source of significant concern. NSAIDs, which are routinely used to treat PDA, can exacerbate jaundice and make the treatment of preterm infants with both conditions challenging.^12,16,29^ The current study showed that the mean value of total bilirubin after treatment in the paracetamol group was 1.38 less than the ibuprofen group, which was also statistically significant (*P* < 0.001). Numerous randomized controlled trials (RCTs) have confirmed the results of this study.^18,29,30^ It has shown that, because of its high affinity to albumin, ibuprofen can displace bilirubin from the albumin site. Furthermore, ibuprofen competes with bilirubin for hepatic glucuronidation. So, potentially the risk of unconjugated hyperbilirubinemia is high with ibuprofen administration in neonates.^3,31^ Contrary to the results of the present study, Desfrere et al showed that the use of NSAIDs did not increase the risk of hyperbilirubinemia.^32^ This discrepancy can be attributed to the near-normal level of bilirubin in the patients who entered their study. Finally, Zecca et al demonstrated that not only did the use of NSIADs in comparison with paracetamol increase bilirubin levels, but it also increased the risk of needing phototherapy and other side effects.^31^ The results of the present study on the effect of drugs on hyperbilirubinemia were similar to the study carried out by El-Mashad et al. Moreover, their study showed that in addition to the risk of hyperbilirubinemia, the risks of gastrointestinal bleeding and increased Cr (renal failure) were decreased with paracetamol treatment.^16^ Unlike El-Mashad and colleagues’ study, the present study did not find a significant difference in Cr levels between the two treatment groups (Tables 3 and 4). Evaluation of Cr levels can play a key role in the prognosis of preterm infants. The discrepancy between the results of the present study and those of El-Mashad and colleagues’ study can be due to the age and birth weight of most of the infants included in the present study. Several studies have evaluated the mediating role of gestational age and birth weight in the effect of NSAID use on the development of renal failure in children.^33,34^ For example, Bagnoli et al reported that taking NSAIDs at a birth age of more than 26 weeks and a birth weight of over 1000 g led to fewer renal effects.^35^ The reason for this observation may be that the kidneys are prostaglandin-dependent organs before 26 weeks, and NSAID use at this age causes renal failure by inhibiting prostaglandin production.^36^ In our study, treatment led to a significant decrease in the rate of Hct response after treatment, such that the mean value of Hct response after treatment in the paracetamol group was 1.65 units less than the ibuprofen group (*P* = 0.005). Since blood sampling is done only according to the protocol in our NICU, lack of significant differences in gestational age and birth weight between the two treatment groups, and considering the fact that critically sick neonates who need more blood sampling were excluded from the study, in the authors’ opinion, this issue cannot be only related to the difference in the frequency of blood sampling between the two treatment groups. Other studies that have examined the laboratory consequences of acetaminophen administration have not reported such a finding. However, some other blood component disorder such as leukopenia, neutropenia, and thrombocytopenia have been noted in the literature as side effects of IV paracetamol in childhood.^37^ In addition, research is ongoing on acetaminophen as a hormone disrupter in the body.^38^ We do not currently have enough knowledge on this subject, but it may be a reason for the presence of acetaminophen side effects.

 The present study has some limitations. The relatively small sample size in this study reduces the study’s power to determine significant changes in clinical and experimental parameters. Another limitation of our study is the lack of classification of the infant’s chronological age at the time of treatment according to gestational age. The current study is a retrospective cohort study; to better determine the efficacy and side effects of drugs, it is necessary to conduct more studies with a higher level of evidence (RCT studies).

 In conclusion, the present study suggested the use of IV paracetamol in preterm neonates who cannot tolerate feeding. In other words, IV paracetamol has equal efficacy with oral ibuprofen in the treatment of PDA; concurrently, IV Paracetamol seems to be associated with lower risk of hyperbilirubinemia following the treatment.
